# Chagas Disease in Ancient Hunter-Gatherer Population, Brazil

**DOI:** 10.3201/eid1406.0707

**Published:** 2008-06

**Authors:** Valdirene S. Lima, Alena M. Iniguez, Koko Otsuki, Luiz Fernando Ferreira, Adauto Araújo, Ana Carolina P. Vicente, Ana Maria Jansen

**Affiliations:** * Oswaldo Cruz Foundation, Rio de Janeiro, Brazil

**To the Editor:** Chagas disease, caused by the protozoan *Trypanosoma cruzi,* and first described by Carlos Chagas in 1909, is endemic to Latin America. As a results of multinational control initiatives launched in the 1990s, the disease prevalence has been reduced. This campaign was focused on the interruption of *T. cruzi* vectorial transmission by eliminating domiciled triatomines. In 2006, Brazil was declared to be free from *T. cruzi* transmission by *Triatoma infestans* (*1*). *T. cruzi* is a heterogeneous taxon with multiple mammal hosts and vectors, besides alternative routes of infection and infective forms. In the Brazilian Amazon region, where domiciled triatomines have not been reported, human cases of Chagas disease have been increasing (*2*). This increase has been attributed to uncontrolled migration and deforestation (*2*). Additionally, recent outbreaks of Chagas disease attributed to oral transmission in previously non–disease-endemic areas out of the Amazon region (*3*) indicate that a new epidemiologic profile is emerging in Brazil.

*T. cruzi* has 2 main genotypes, *T. cruzi* I and *T. cruzi* II, and these subpopulations display distinct biologic, biochemical, and genetic profiles. In Brazil, *T. cruzi* I is widespread among wild mammals and sylvatic vectors of all biomes. Moreover, this genotype is commonly isolated from humans and wild mammals in the Amazon Basin. In contrast, *T. cruzi* II, has a focal distribution in nature but is the main agent of human infection in other Brazilian regions (*4*).

In this report, we describe the finding of *T. cruzi* in human remains dating back 4,500–7,000 years that were obtained from a Brazilian archeological site and, the recovery of an ancient DNA (aDNA) sequence corresponding to the parasite lineage type I. The mummy, called AM1, was a woman ≈35 years of age from a hunter-gatherer population. She was found in Abrigo do Malhador archeological site, Peruaçu Valley, Minas Gerais State (*5*). This region, where the semiarid ecosystem is predominant, has a dry climate, karst relief (an area of limestone terrain characterized by sinks, ravines, and underground streams), and soil with a basic pH. These conditions have contributed to the preservation of specimens.

The remains were excavated in 1985 and maintained in an environment protected from light and humidity. In 2005, after taking precautions to avoid contamination with exogenous DNA or cross-contamination between samples, we collected ≈6 cm of a rib fragment from AM1. All experiments were conducted in a restricted area that was isolated from the major laboratory, where post-PCR experiments were performed. *T. cruzi* had never been used in either laboratory. Mitochondrial DNA haplotypes of laboratory staff were determined to control contamination. PCR-positive controls (*T. cruzi*) were not used. The rib surface was gently scraped to remove impurities and decontaminated with bleach (6% NaOCl) and UV light (15 min for each side). We processed 300 mg of bone powder according to the extraction protocol of dehybernation B solution of the Geneclean Kit for Ancient DNA (Bio101, La Jolla, CA, USA). By using specific set of primers (*6*), a 350-bp miniexon gene fragment was successfully recovered by PCR (Figure); the fragment corresponded to the *T. cruzi* I lineage, according to miniexon gene typing assay (*6*). Moreover, nucleotide sequence analysis (341 bp) (GenBank accession no. EF626693), showed 98% similarity with *T. cruzi* I sequences in GenBank. Additionally, total aDNA hybridization with *T. cruzi* probes (miniexon and total kinetoplast DNA) confirmed the infection. Sequence analysis of the human mitochondrial DNA HVS-I region characterized this person as belonging to haplogroup B (GenBank accession no. EU359272), one of the founder human haplogroups in the Americas.

**Figure:**
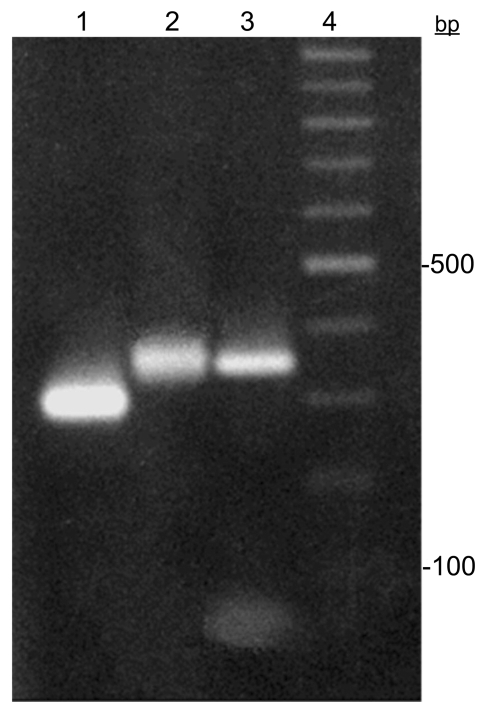
*Trypanosoma cruzi* miniexon gene typing assay. Lanes 1 and 2, *T. cruzi* II (300 bp) and *T. cruzi* I (350 bp) markers, respectively; lane 3, *T. cruzi* I cloned fragment recovered from Brazilian mummy; lane 4, 100-bp ladder.

The antiquity of human *T. cruzi* infection in South America has been demonstrated on the basis of paleonthologic studies. Clinical manifestations of Chagas disease were observed in Chilean mummies from pre-Columbian times (*7*). Moreover, a *T. cruzi* kinetoplast DNA region was recovered in Chilean and Peruvian mummies from up to 9,000 years ago (*8*,*9*).

In Brazil, the current epidemiologic scenario concerning Chagas disease in indigenous populations involves ecologic aspects of their settlements, along with nomad habits, which prevent triatomine nesting and, therefore, the infection. The beginning of *T. cruzi* transmission to humans is attributed to the domiciliation of *T. infestans* as a consequence of precarious mud dwellings, built after European colonization (*10*). In this report, we showed that *T. cruzi* human infection in Brazil is ancient, dating back at least 4,500 years, and therefore occurring in hunter-gatherer populations largely preceding *T. infestans* domiciliation. The presence of the *T. cruzi* I genotype infecting humans 4,500–7,000 years ago in Minas Gerais State, where this genotype is currently absent (*6*), suggests that the distribution pattern of *T. cruzi* genotypes in humans has changed in time and place. Moreover, the recovery of an aDNA sequence and the possibility of genotyping parasites from human remains make it possible to reconstruct the early dispersion patterns of *T. cruzi* subpopulations. On the basis of our results, one may speculate that the current outbreaks of human *T. cruzi* infection, independent of triatomine domiciliation, are the reemergence of the ancient epidemiologic scenario of Chagas disease in Brazil.
